# Copaiba Oil: An Alternative to Development of New Drugs against Leishmaniasis

**DOI:** 10.1155/2012/898419

**Published:** 2011-06-12

**Authors:** Adriana Oliveira dos Santos, Tânia Ueda-Nakamura, Benedito Prado Dias Filho, Valdir Florêncio da Veiga Junior, Celso Vataru Nakamura

**Affiliations:** ^1^Programa de Pós-Graduação em Microbiologia, Universidade Estadual de Londrina, Rodovia Celso Garcia Cid s/n, 86051-990 Londrina, PR, Brazil; ^2^Programa de Pós-Graduação em Ciências Farmacêuticas, Laboratório de Inovação Tecnológica no Desenvolvimento de Fármacos e Cosméticos, Universidade Estadual de Maringá, Bloco B-08, Avenida Colombo 5790, 87020-900 Maringá, PR, Brazil; ^3^Departamento de Química, Instituto de Ciências Exatas, Universidade Federal do Amazonas, Avenida General Rodrigo Octávio Jordão Ramos 3000, Japiim, 69077-000 Manaus, AM, Brazil

## Abstract

Leishmaniasis is a neglected disease that is increasing globally at an alarming rate. Glucantime has been the therapy of choice for more than 50 years. A recent study reported the antileishmanial activity of copaiba oil against *Leishmania amazonensis*. These results led us to investigate morphological and ultrastructural changes in *L. amazonensis* treated with copaiba oil, using electron microscopy and flow cytometry to assess specific organelles as targets for copaiba oil. In the promastigote and axenic amastigote forms, this copaiba oil caused notable morphological and ultrastructural changes, including extensive mitochondrial damage and denaturation of the plasma membrane. Copaiba oil treatment also induced a decrease in Rh123 fluorescence, suggesting interference with the mitochondrial membrane potential and loss of cell viability with an increase in plasma membrane permeability, as observed by flow cytometry after staining with propidium iodide. In conclusion, copaiba oil could be exploited for the development of new antileishmanial drugs.

## 1. Introduction

Parasites of the genus *Leishmania* cause a wide spectrum of human infections, ranging from the disfiguring mucosal and cutaneous forms of the disease to the life-threatening visceral form [1–4]. The species and/or strains of the *Leishmania* parasites strongly influence the clinical features of leishmaniasis, including host immunity [5]. There are approximately 21 species of *Leishmania*, transmitted by about 30 phlebotomine sandfly species [6]. The number of cases of leishmaniasis is increasing globally at an alarming rate. According to the World Health Organization, this group of diseases is endemic in 88 countries in Africa, Asia, Europe, and North and South America, with a total of 350 million people at risk [7]. Despite its increasing worldwide incidence, leishmaniasis has become one of the so-called neglected diseases, with little interest by financial donors, public-health authorities, and professionals to implement activities to research, prevent, or control the disease [8–10]. During a complex digenetic life cycle, flagellated *Leishmania* parasites alternate between promastigote (living in the gut of their sandfly vector) and amastigote forms (living in their mammalian host), which differ significantly in cellular morphology and flagellum length [11]. The parasites have developed a variety of adaptive mechanisms to evade the vertebrate host immune responses, including survival within the host macrophage [12, 13]. Because leishmaniasis mainly affects poor countries, research and development of new drugs have been seriously neglected [6, 8, 10, 14]. The pentavalent antimonials (Glucantime) have been the therapy of choice for more than 50 years. Although the lipid formulations of amphotericin B are an important advance in therapy, their high cost precludes their use [15]. Consequently, there is an urgent need to discover new drugs that are effective against leishmaniasis. Plants are very promising subjects, since they have been important sources of substances with many therapeutic uses [16–18]. In addition, the American flora is one of the world's wealthiest sources of material with pharmacological activity, due to its biodiversity [19]. Copaiba oil has been used in folk medicine since the 19th century [19, 20]. The use of copaiba oils to treat leishmaniasis is cited in several ethnopharmacological studies [21–24]. A recent study by Santos et al. [25] found that copaiba oils obtained from different species of *Copaifera *show activity against promastigote forms of *L. amazonensis*. Significant antileishmanial activity of copaiba oil from *C. reticulata* was demonstrated against axenic amastigote and intracellular amastigote forms of the parasite. These findings led us to investigate the morphological and ultrastructural changes in *L. amazonensis* treated with copaiba oil, using electron microscopy and flow cytometry to investigate specific organelles as targets for copaiba oil. 

## 2. Materials and Methods

### 2.1. Plant Material

*Copaifera reticulata *Ducke was collected in Belém, state of Pará, and deposited in the Herbarium of the Instituto Nacional de Pesquisas da Amazônia (INPA, Manaus) under number INPA 61,212.

### 2.2. Parasites

*Leishmania amazonensis *MHOM/BR/ 75/Josefa strain was isolated from a patient with diffuse cutaneous leishmaniasis by Cesar Augusto Cuba-Cuba (Universidade de Brasília, Brazil). Promastigote forms were cultured at 25°C in Warren's medium (brain-heart infusion plus haemin and folic acid), pH 7.0, supplemented with 10% heat-inactivated foetal bovine serum (FBS) (Gibco Invitrogen Corporation, New York, USA), in a tissue flask with weekly transfers. Axenic amastigote cultures obtained by *in vitro* transformations of infective promastigotes [26] were incubated at 32°C in Schneider's insect medium (Sigma Chemical Co., St. Louis, MO, USA), pH 4.6, with 20% foetal bovine serum. 

### 2.3. Scanning Electron Microscopy

The parasites were treated with concentrations of copaiba oil from *C. reticulata* that inhibited 50% and 90% of growth (IC_50_ and IC_90_, resp.). Promastigotes were treated with 5.0 and 80.0 *μ*g/mL at 25°C, and axenic amastigote forms were treated with 15.0 and 70.0 *μ*g/mL at 32°C. Next, the parasites were collected by centrifugation after 72 h incubation, washed in PBS buffer, fixed with 2.5% glutaraldehyde in 0.1 M sodium cacodylate buffer containing 1.0 mM CaCl_2_. After fixation, small drops of the sample were placed on a specimen support with poly-L-lysine. The samples were then dehydrated in graded ethanol, critical-point dried in CO_2_, coated with gold, and observed in a Shimadzu SS-550 scanning electron microscope. 

### 2.4. Transmission Electron Microscopy

Promastigote and axenic amastigote forms of *L. amazonensis* were treated with IC_50_ and IC_90_ of copaiba oil. After that, the samples were processed for transmission electron microscopy. Parasite cells were harvested and washed twice with PBS buffer, and fixed with 2.5% glutaraldehyde in 0.1 M sodium cacodylate buffer at 4°C, postfixed in a solution containing 1% OsO_4_, 0.8% potassium ferrocyanide, and 10 mM CaCl_2_ in 0.1 M cacodylate buffer, dehydrated in an increasing acetone gradient, and embedded in Epon resin. Next, ultrathin sections were stained with uranyl acetate and lead citrate, and images were obtained in a Zeiss 900 TEM. 

### 2.5. Measurement of Mitochondrial Membrane Potential

The mitochondrial membrane potential was measured in *L. amazonensis* axenic amastigotes using Rhodamine 123 (Rh 123) reagent, following the manufacturer's protocol. Briefly, axenic amastigotes (5 × 10^6^ parasites/mL) after treatment with copaiba oil from *C. reticulata *(100, 150, 200 *μ*g/mL for 3 h at 32°C) or untreated cells were harvested and washed with PBS buffer. After that, the cells were incubated with Rh 123 (5 mg/mL for 30 min at 37°C) and washed twice with PBS buffer. The mean fluorescence intensity was analysed using FACSCalibur and CellQuest software. A total of 10,000 events were acquired in the region established as that corresponding to the parasites. The compound carbonyl cyanide m-chlorophenylhydrazone (CCCP) was used as a positive control. 

### 2.6. Measurement of Cell Viability

Cell viability was checked by staining the cells with propidium iodide (PI). Axenic amastigotes treated with copaiba oil from *C. reticulata *(100 and 200 *μ*g/mL for 3 h at 32°C) or untreated amastigotes were harvested and washed with PBS buffer. Then, the parasite cells were stained with PI (20 *μ*g/mL for 5 min) as per instructions given by the manufacturer. The mean fluorescence intensity of the cells was analysed by flow cytometry, using FACSCalibur and CellQuest software. A total of 10,000 events were acquired in the region established as that corresponding to the parasites. Amphotericin B was used as a positive control. 

### 2.7. Statistical Analysis

The comparison between the values of fluorescence for Rh123 and PI was performed with the program GraphPad Prism 4 (GraphPad Software, San Diego, CA, USA). Student's *t-*test was applied, and a *P*-value less than .05 was regarded as significant. The means and standard deviations were determined from at least three experiments. 

## 3. Results and Discussion

Copaiba oils obtained from different species of *Copaifera *showed activity against promastigote forms of *L. amazonensis*. Significant antileishmanial activity of copaiba oil from *C. reticulata* was demonstrated against axenic amastigote and intracellular amastigote forms of the parasite with IC_50_ values of 15.0 *μ*g/mL and 20.0 *μ*g/mL, respectively. As reported recently, the major component of *C. reticulata* is *β*-caryophyllene (40.9%) [25]. Therefore, the antileishmanial activity of *β*-caryophyllene was tested against all forms of the parasite. However, *β*-caryophyllene did not show antileishmanial activity against amastigote-like forms, compared with copaiba oil (data not shown). Some investigators have attributed the biological activities of copaiba oil to the complex nature of the sesquiterpene and diterpene mixture, which might affect the active component by a synergistic effect [27]. Additionally, Lima et al. [28] reported that fractionation of copaiba oils results in fractions that are less active than the crude oil itself.

**Figure 1:**
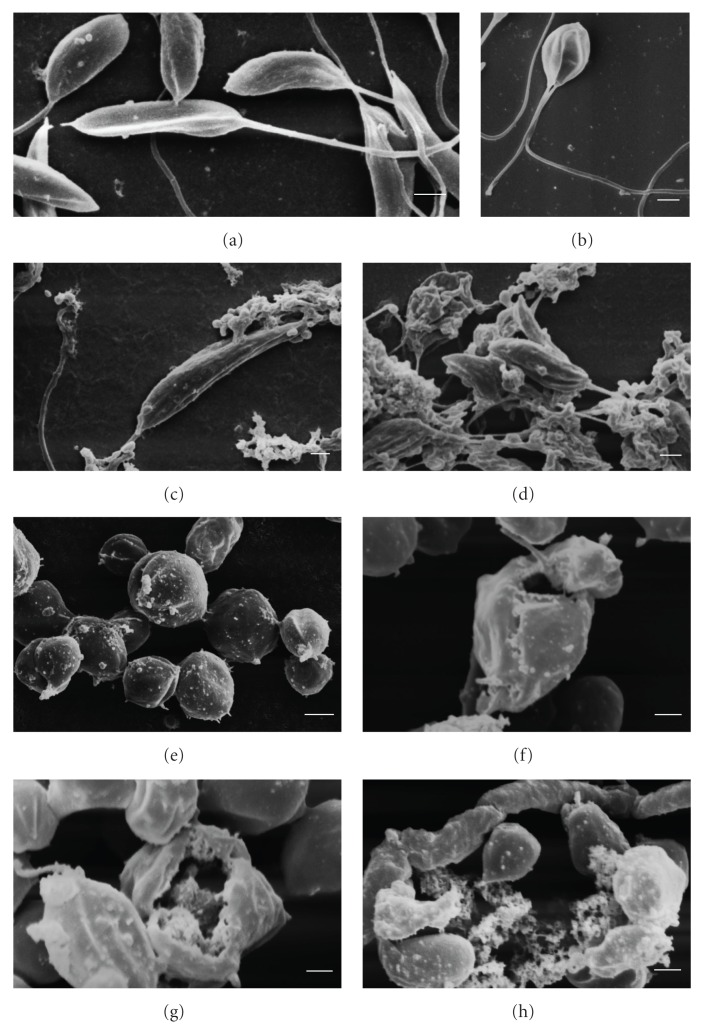
Scanning electron micrographs of promastigote and axenic amastigote forms of *L. amazonensis* treated with copaiba oil for 72 h. (a) promastigote, control; (b) promastigote after treatment with IC_50_ of copaiba oil; (c and d) promastigote after treatment with IC_90_ of copaiba oil; (e) amastigote, control; (f) amastigote after treatment with IC_50_ of copaiba oil; (g and h) amastigote after treatment with IC_90_ of copaiba oil. Bars = 1 *μ*m.

**Figure 2:**
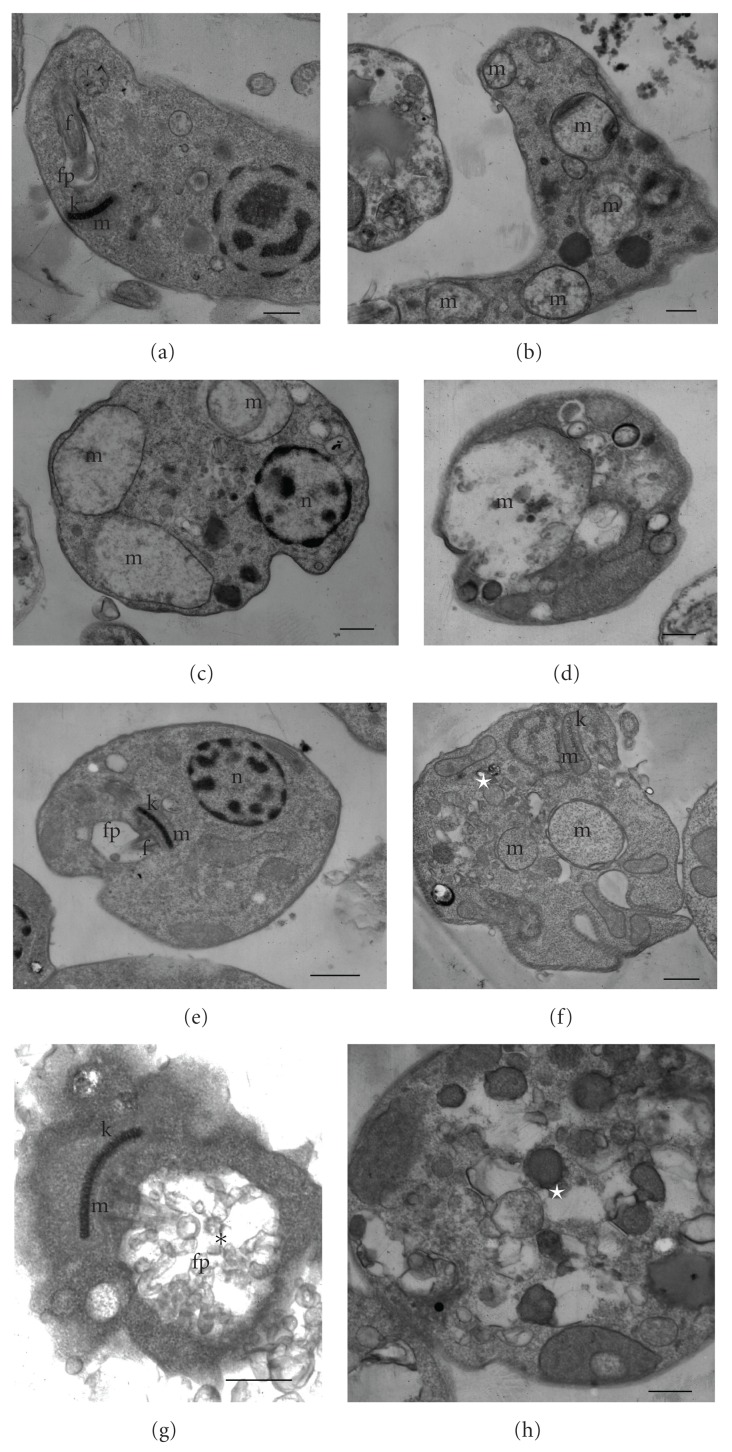
Ultrastructural effect of copaiba oil after incubation for 72 h on promastigote and axenic amastigote forms of *L. amazonensis, *observed by transmission electron microscopy. (a) promastigote control; (b) promastigote treated with IC_50_ of copaiba oil; (c and d) promastigote treated with IC_90_ of copaiba oil; (e) amastigote, control; (f) amastigote treated with IC_50_ of copaiba oil; (g and h) amastigote treated with IC_90_ of copaiba oil. Bars = 1 *μ*m. The treatment with copaiba oil led to changes in the mitochondria (m), exocytic activity in the region of the flagellar pocket (asterisk), and cytoplasmic vacuolisation (white star). n, nucleus; f, flagellum; fp, flagellar pocket; k, kinetoplast; m, mitochondrion.

**Figure 3:**
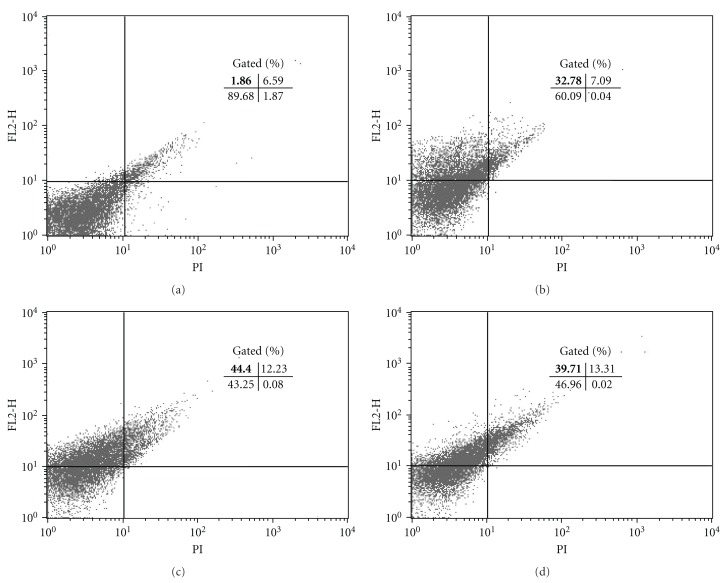
Flow cytometry analysis of *L. amazonensis* axenic amastigotes treated with copaiba oil and stained with propidium iodide (PI), showing a decrease of cell viability in treated cells, (a) Untreated cells (b) amphotericin-B; (c) amastigotes treated with 100 *μ*g/mL; (d) amastigotes treated with 200 *μ*g/mL. The numbers in bold show the percentage of PI-stained positive cells in the upper left quadrant.

**Figure 4:**
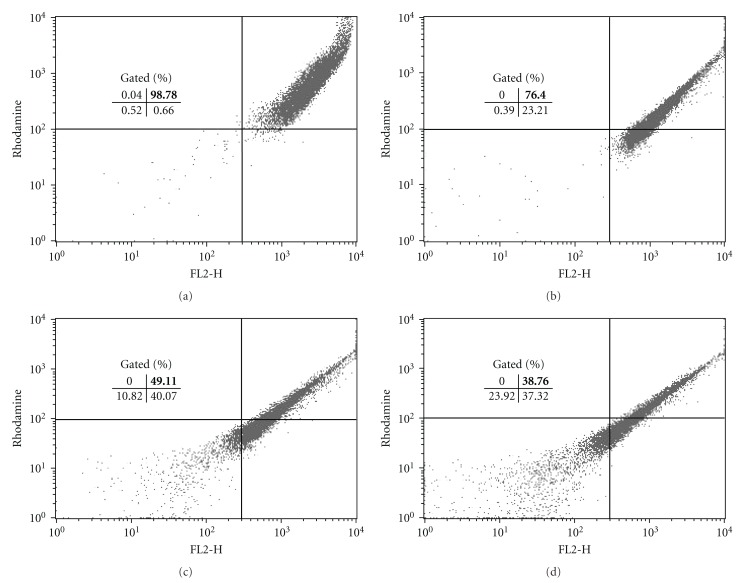
Flow cytometry analysis of Rh123-labeled axenic amastigotes of *L. amazonensis*. Copaiba oil collapsed the ΔΨ_m_, leading to parasite death. (a) untreated cells; (b) CCCP 200 *μ*M; (c) amastigotes treated with 100 *μ*g/mL; (d) amastigotes treated with 200 *μ*g/mL. The numbers in bold represent the percentage of collapsed ΔΨ_m_ cells in the upper right quadrant.

These results led us to investigate the morphological and ultrastructural changes in *L. amazonensis* treated with copaiba oil from *C. reticulata, *using electron microscopy. As observed by scanning electron microscopy, promastigote and axenic amastigote forms treated with copaiba oil showed notable morphological changes (Figure 1). Untreated control promastigotes showed the typical elongated shape with a single flagellum (Figure 1(a)), and axenic amastigotes a rounded shape (Figure 1(e)). The typical morphology of promastigotes and axenic amastigotes changed drastically after treatment with copaiba oil. Promastigote forms treated with copaiba oil showed a rounded shape with two flagella (Figure 1(b)). In amastigote-like forms, treatment with copaiba oil led to rupture of the plasma membrane with loss of their contents (Figures 1(f) and 1(g)). Additionally, both forms showed protein denaturation of the cell surface (Figures 1(c), 1(d), 1(g), and 1(h)). Several studies have found that the cell surface (carbohydrates associated with lipids to form glycolipids) of parasitic protozoa plays an important role in various processes including cell recognition, cell adhesion, regulation of cell growth, expression of surface antigens, and receptors [29–31]. Consequently, surface changes caused by copaiba oil treatment may affect the parasite-host interaction, decreasing the infectivity of the parasite. After treating the intracellular amastigotes with copaiba oil from *C. reticulata, *Santos et al. [25] observed a dose-dependent decrease of parasites in the host peritoneal macrophages. The cell viability of axenic amastigotes of *L. amazonensis* was checked by staining the cells with propidium iodide (PI), a fluorescent dye that binds specifically to DNA. In untreated axenic amastigotes, the degree of binding of PI was 1.86%. Following treatment of axenic amastigotes with copaiba oil at 100 *μ*g/mL and 200 *μ*g/mL for 3 h, the gated percentage of PI-stained cells increased to 44.4% and 39.7%, respectively (Figure 3, upper-left quadrant). These results were similar to the positive control amphotericin B (32.78% treated with 5 *μ*g/mL; Figure 3, upper-left quadrant). All treatments were applied for 3 h at 32°C. Student's *t-*test (*P* < .05) indicated significant differences between the group treated with copaiba oil compared to the negative control group. These data may indicate that in axenic amastigotes, copaiba oil induces a considerable increase in plasma-membrane permeability. Ultrastructural changes in promastigote and axenic amastigote forms of *L. amazonensis* treated with the copaiba oil from* C. reticulata* are illustrated in Figure 2. Control cells (Figures 2(a) and 2(e)) showed no plasma membrane alterations, a nucleus with a normally centred nucleolus, a kinetoplast, a mitochondrion with well-defined mitochondrial crypts, and a flagellar pocket with only one flagellum, or the short flagellum of the amastigote-like form is within a distended flagellar pocket. In both the promastigote and amastigote-like forms, the most prominent effect of the treatment was swollen mitochondria (Figures 2(b), 2(c), 2(d), and 2(f)). The copaiba oil treatment also induced intense exocytic activity in the region of the flagellar pocket (Figure 2(g)) and cytoplasmic vacuolisation (Figure 2(h)). Alterations in the mitochondrial membrane potential of *L. amazonensis* amastigotes were studied by treatment with copaiba oil for 3 h at 32°C and stained with Rh-123 as described in Methods. Mitochondrial energizing induces quenching of Rh-123 fluorescence, and the rate of fluorescence decay is proportional to the mitochondrial membrane potential. Data obtained from flow cytometry by using Rh123 showed a marked decrease in the percentage population of the upper right gated (49.11% and 38.76%), indicating depolarization of the mitochondrial membrane potential in the cells following treatment with copaiba oil at 100 *μ*g/mL and 200 *μ*g/mL, respectively (Figure 4). Similarly, a decrease in membrane potentials was also observed following treatment with the standard drug carbonyl cyanide m-chlorophenylhydrazone (CCCP) (76.4%) at 200 *μ*M for 3 h at 32°C. In contrast, untreated cells maintained the membrane potential (98.78%) (Figure 4; upper right quadrant). Student's *t-*test (*P* < .05) indicated significant differences between cells treated copaiba oil compared to the negative control group. The maintenance of mitochondrion properties, including the mitochondrial membrane potential (ΔΨ_m_), synthesis of ATP, and oxidative phosphorylation, is essential for the survival of *Leishmania*, because these parasites have a single mitochondrion [32–34]. Accordingly, the loss of the mitochondrial membrane potential is one indication of damage observed in the ultrastructural analyses and might lead to the death of the parasites.

In conclusion, the copaiba oil caused notable morphological and ultrastructural changes in the treated parasites compared with the untreated cells. In both forms of *L. amazonensis*, copaiba oil caused extensive mitochondrial damage and also led to plasma membrane denaturation. Since morphological and ultrastructural analysis demonstrated that treatment with copaiba oil induced alterations in the membrane and mitochondrion of the parasites, we incubated treated parasites with PI and Rh123. *L. amazonensis *treated with copaiba oil showed a decrease in Rh123 fluorescence, suggesting an effect on the mitochondrial membrane potential. Staining of the parasite cells with PI demonstrated that loss of cell viability occurred due to an increase in permeability of the plasma membrane. Therefore, it appears that copaiba oil could be exploited for the development of new antileishmanial drugs.
